# Insight into the role of the gut-brain axis in alcohol-related responses: Emphasis on GLP-1, amylin, and ghrelin

**DOI:** 10.3389/fpsyt.2022.1092828

**Published:** 2023-01-09

**Authors:** Maximilian Tufvesson-Alm, Olesya T. Shevchouk, Elisabet Jerlhag

**Affiliations:** Department of Pharmacology, Institute of Neuroscience and Physiology, The Sahlgrenska Academy, University of Gothenburg, Gothenburg, Sweden

**Keywords:** appetite-regulatory peptides, addictive drugs, dependence, reward, dopamine

## Abstract

Alcohol use disorder (AUD) contributes substantially to global morbidity and mortality. Given the heterogenicity of this brain disease, available pharmacological treatments only display efficacy in sub-set of individuals. The need for additional treatment options is thus substantial and is the goal of preclinical studies unraveling neurobiological mechanisms underlying AUD. Although these neurobiological processes are complex and numerous, one system gaining recent attention is the gut-brain axis. Peptides of the gut-brain axis include anorexigenic peptide like glucagon-like peptide-1 (GLP-1) and amylin as well as the orexigenic peptide ghrelin. In animal models, agonists of the GLP-1 or amylin receptor and ghrelin receptor (GHSR) antagonists reduce alcohol drinking, relapse drinking, and alcohol-seeking. Moreover, these three gut-brain peptides modulate alcohol-related responses (behavioral and neurochemical) in rodents, suggesting that the alcohol reduction may involve a suppression of alcohol’s rewarding properties. Brain areas participating in the ability of these gut-brain peptides to reduce alcohol-mediated behaviors/neurochemistry involve those important for reward. Human studies support these preclinical studies as polymorphisms of the genes encoding for GLP-1 receptor or the ghrelin pathway are associated with AUD. Moreover, a GLP-1 receptor agonist decreases alcohol drinking in overweight patients with AUD and an inverse GHSR agonist reduces alcohol craving. Although preclinical and clinical studies reveal an interaction between the gut-brain axis and AUD, additional studies should explore this in more detail.

## 1. Introduction

### 1.1. Alcohol use disorder

Alcohol use disorder (AUD) contributes substantially to the world-wide mortality and morbidity (1). Indeed, harmful alcohol use is associated with approximately 5% of all deaths and contributes to over 200 diseases. Beyond these negative health consequences, it contributes to socioeconomical losses for both society and individuals (2, 3). It is a relapsing brain disease characterized by reoccurring phases of craving, loss of control and an escalated intake over time. The AUD cycle involves three central stages, characterized by different behaviors, that are repeated over time [for review see (4)]. In the initial binge part of AUD, reward mediated by the mesolimbic dopamine system is crucial. This reward associated system consists of dopaminergic neurons of the ventral tegmental area (VTA) that projects to areas like nucleus accumbens (NAc) or amygdala. This neurocircuit also appears central for the second part of the AUD cycle, namely compulsive alcohol-taking. The third stage of the repeated AUD cycle is the consumption of alcohol due to an avoidance of negative and abstinence symptoms [for review see (4)]. During abstinence patients with AUD experience craving, another feature where the mesolimbic dopamine system participates. In summary, this suggests that the rewarding properties of alcohol is one important aspect underlying AUD process. In agreement, the alcohol’s rewarding experience has been identified as a risk factor for later AUD diagnosis (5).

For a multifaceted disorder like AUD, one animal model cannot be used to reflect its complexity, but can rather be used together to reflect aspects thereof [for extensive review see (6)]. In various alcohol drinking paradigms alcohol intake, binge drinking and an escalation over time can be observed. Withdrawal of alcohol causes relapse drinking, which has been suggested to reflect craving in a human situation. Moreover, this withdrawal causes abstinence symptoms in rodents as it does in humans. In the operant self-administration model aspects like alcohol consumption, the motivation to consume alcohol and alcohol-seeking can be studied. In humans the reward of alcohol is positively associated with dopamine release in nucleus accumbens (NAc), and similarly alcohol releases dopamine in NAc in rodents [for review see (7)]. Therefore, preclinical models like locomotor activity, and dopamine release in NAc are used as they reflect activation of the mesolimbic system and tentatively reward. Moreover, the conditioned place preference (CPP) test can be used to reflect either alcohol reward (rCPP) or the memory of the alcohol-induced reward (mCPP).

These preclinical models have been used in attempts to define the complex pathophysiology of the AUD process, where the multifaceted neurobiological processes of each of these stages has to be studied and various players have been defined. Intriguingly, the underpinnings of AUD stages involve multiple signals that may diverge and overlap to some extent. Collectively such studies have contributed to the approval of AUD medications. Today, four pharmaceuticals with different mechanism of action have been approved for treatment of AUD: Disulfiram, acamprosate, naltrexone, and nalmefene. Importantly, clinical studies reveal a reduction in alcohol drinking in AUD patients by these agents (8–10). However, the heterogeneity of a complex disease like AUD contributes to the limited efficacy of these pharmaceuticals [for review see (1, 11)] and thus additional treatments are warranted; an aim of studies exploring the neurobiological substrates of AUD. These neurobiological underpinnings have been characterized extensively and recent studies imply the gut-brain axis as an important modulator of the AUD cycle [for review see (12)].

### 1.2. The gut-brain peptides glucagon-like peptide-1, amylin, and ghrelin

The role of the gut-brain axis in maintaining glucose and energy homeostasis is crucial and involve a number of different peptides (13). Although all these peptides display important physiological and behavioral effects, glucagon-like peptide-1 (GLP-1), amylin, and ghrelin have gained extra interest as they play an important role for the regulation of alcohol responses. It should, however, be noted that other important gut-brain peptides have been studied in relation to alcohol, and the importance of the neuropeptide orexin and galanin has been reviewed elsewhere [for review see (14, 15)].

#### 1.2.1. Glucagon-like peptide-1

Preproglucagon (PPG) containing cells/neurons of the intestine, pancreas, nucleus of the solitary tract (NTS), and olfactory bulb produce GLP-1 (16, 17). It is secreted after a meal to induce satiation, and is thereafter rapidly degraded by DPP-IV and neutral endopeptidase 24.11 [for review see (17)]. GLP-1 acts *via* its receptor, GLP-1R, to regulate a wide range of physiological properties. Of these, its ability to normalize plasma glucose levels though a facilitation of insulin secretion (18) led to the approval of GLP-1 related treatments for type II diabetes (19). Moreover, the ability of GLP-1R agonists to reduce feeding, appetite, and body weight gain (20–27) have contributed to the approval of these compounds to treat obesity [for review see (28)]. Due to the above-mentioned quick degradation, long-acting GLP-1R agonists have been developed [see (29)]. Exendin-4 (Ex-4)/exenatide and liraglutide are injected twice daily or daily respectively, whereas dulaglutide, and semaglutide are used as once weekly treatments. Another way to stimulate the GLP-1 pathway is to enhance the circulating levels of GLP-1 by inhibition of DPP-IV, by using the diabetic agents sitagliptin and linagliptin.

#### 1.2.2. Amylin

Another gut-brain peptide produced in the pancreas is amylin, which is co-secreted with insulin and works synergistically with insulin to decrease blood glucose. The amylin analog pramlintide is thus approved for the treatment of diabetes [for review see (30)]. Besides this effect on glucose homeostasis, amylin reduces homeostatic and hedonic feeding, causes satiety, and reduces body weight [for review see (30)]. Amylin related compounds are thus being tested as anti-obesity agents. Amylin acts *via* the amylin receptor (AMYR), which consists of the calcitonin receptor (CTR) together with one of three receptor activity-modifying proteins (RAMP1-3). To date, preclinical studies mostly use amylin or salmon calcitonin (sCT; an AMYR agonist) when investigating behavioral roles associated with the amylin pathway (31).

#### 1.2.3. Ghrelin

Ghrelin (acyl-ghrelin) is an orexigenic peptide, with the ability to increase both the hedonic and homeostatic aspects of feeding behaviors and is released pre-prandially to stimulate appetite and hunger [for review see (32)]. Therefore, suppression of the ghrelin pathway has been suggested as one way to treat obesity. However, no available ghrelin related treatments exist clinically and therefore antagonists like JMV2969 and [D-Lys3]-GHRP-6 are used only in research. Besides feeding, ghrelin controls multiple physiological properties such as growth hormone release, cardiovascular function and gut motility [for review see (32)]. These effects are initiated *via* the activation of growth hormone secretagog receptor (GHSR, ghrelin receptor), a G-protein coupled receptor with ligand-independent abilities. GHSR have a high intrinsic activity, can form heterodimers and are allosterically modulated by other receptors [for review see (32)]. Ghrelin is mainly produced and secreted in the stomach and intestine, but additional production may exist in brain regions like hypothalamus [for review see (32)].

## 2. The role of glucagon-like peptide-1, amylin, and ghrelin on alcohol-related responses in animals and humans

**FIGURE 1:**
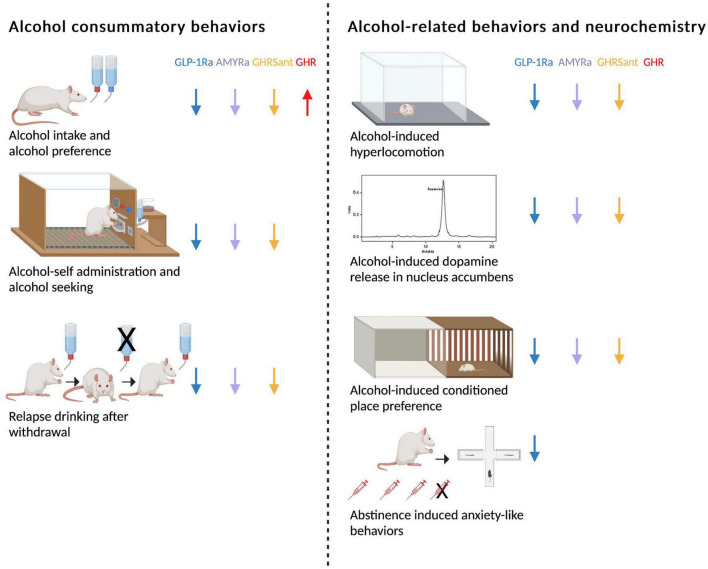
Schematic summary of how glucagon-like peptide-1 receptor agonists (GLP-1Rant), amylin receptor agonists (AMYRa), ghrelin receptor antagonists (GHSRant), and ghrelin (GHR) influence alcohol consummatory behaviors and alcohol-related behaviors and neurochemistry in rodents (↓; decrease, ↑; increase). Created by BioRender.com.

**FIGURE 2:**
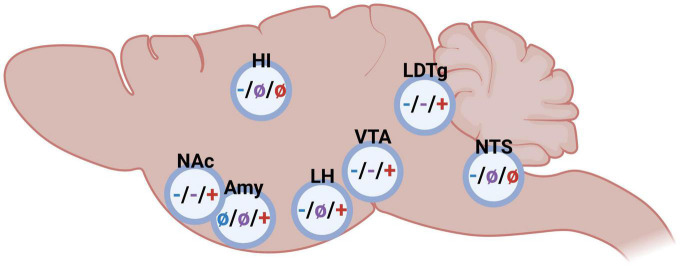
Schematic illustrations on the impact of glucagon-like peptide-1 (blue), amylin (purple), and ghrelin (red) in different brain regions on alcohol-related behaviors in rodents (– inhibition/decrease; + stimulation/increase, not studied). Nucleus accumbens (NAc), amygdala (Amy), hippocampus (HI), lateral hypothalamus (LH), ventral tegmental area (VTA), laterodorsal tegmental area (LDTg), and nucleus of the solitary tract (NTS). Created by BioRender.com.

As mentioned above, GLP-1, amylin and ghrelin are well-known for their effects on feeding behaviors. However, the expression of their receptors is wide-spread and include areas associated with reward (16, 33–40). This review will discuss more recent findings showing that these three peptides modulate the response to rewards like alcohol in animals and humans. First, their effect on different alcohol consummatory behaviors is summarized, and then their ability to influence alcohol-related responses (behavior and neurochemistry) is introduced (Figure 1). Thereafter, brain regions participating in this interaction is reviewed (Figure 2). On a final note, available human studies addressing this interaction are presented. In each segment below, the findings from the GLP-1 system will be presented first, followed by amylin and finally data related to ghrelin signaling; a comparison is missing in available reviews.

### 2.1. The role of glucagon-like peptide-1, amylin, and ghrelin on alcohol consummatory behaviors

#### 2.1.1. Glucagon-like peptide-1

The ability of different GLP-1R agonists to reduce alcohol drinking has been shown in a vast number of preclinical studies (Figure 1). In male rats exposed to alcohol for long periods of time, acute treatment with either Ex-4, GLP-1, liraglutide or semaglutide reduces both the alcohol consumption and the preference for alcohol (41–45). A reduction is also evident in non-human primates treated with exenatide or liraglutide (46). In line with the findings after acute administration, repeated injections of liraglutide decreases alcohol intake in male rats (44). Moreover, weekly treatment with dulaglutide for 5–9 weeks reduces the consumption of and preference for alcohol in male and female rats throughout the entire treatment period (47). Although the decrease in alcohol drinking is slightly less pronounced in female compared to male rats, this is the first study demonstrating a decline in female rats (47). The findings that dulaglutide treatment does not cause a tolerance and the lowered alcohol drinking persists after treatment discontinuation (44, 47, 48), may be beneficial in a clinical situation. In group-housed male mice Ex-4 treatment decreases alcohol drinking, most likely due to an altered drinking pattern. Specifically, the latency to first drinking Is enhanced and the number of drinks is lowered after Ex-4 (49). The ability of GLP-1R agonists to reduce alcohol drinking may be associated with both central and peripheral GLP-1R as the expression of GLP-1R exists throughout the body. However, the profound decline in alcohol drinking by the different GLP-1R agonists appear to depend on GLP-1R in the brain rather than body (50).

Taken together these preclinical studies show that GLP-1R activation is required for alcohol consumption, an effect persistent across species and GLP-1R agonists used. However, there are different ways to target the GLP-1 pathway, where two alternatives are stimulation of the receptor or the enhancing endogenous GLP-1 levels. Compared to GLP-1R activation, the role of the peptide itself for alcohol drinking is less studied. However, a few studies report that manipulation of GLP-1 centrally (42, 51) but not peripherally (45) alters alcohol drinking. It should thus be suggested that the detailed role of GLP-1 and the origin thereof is a tentative focus on up-coming studies.

Besides controlling alcohol consumption as summarized above, GLP-1R activation influences the motivation to consume alcohol and relapse drinking observed after alcohol withdrawal. On this note, in male rats the motivation to consume alcohol in the operant self-administration model is lowered by Ex-4 acutely or liraglutide repeatedly (41, 44, 52). Similarly, in this model Ex-4 suppresses the progressive ratio for alcohol in male rats, indicating that Ex-4 reduces alcohol-seeking (41). Additional preclinical studies show that two different GLP-1R agonists (Ex-4 or AC3174) prevent relapse drinking in male rodents (44, 48, 49). Abstinence symptoms is another AUD criteria, that may be influenced by the GLP-1 pathway in rodents; both the receptor and the peptide itself. Specifically, abstinence symptoms during withdrawal such as anxiety is prevented by liraglutide (the GLP-1R agonist) or sitagliptin (a DPP-IV inhibitor that enhances endogenous GLP-1) (53, 54). Although preclinical models may not signify AUD diagnosis, they may model aspects thereof. Therefore, together these data may indicate that GLP-1R regulate different aspects of the AUD cycle.

#### 2.1.2. Amylin

In contrast to the role of the GLP-1 pathway for alcohol consumption behaviors, the influence of AMYR thereof is less studied (Figure 1). It was initially demonstrated that acute or repeated treatment of sCT (AMYR agonist) lowers alcohol intake in high alcohol-preferring rats exposed to alcohol for prolonged period of times (55, 56). Similarly, another AMYR agonist, AMY1213, decreases alcohol drinking in both male and female rats with long alcohol drinking before treatment (57). These latter findings display for the first time that both sexes respond to AMYR activation, however, the response is more beneficial in females. The modulatory role of AMYR on alcohol drinking is further supported as the AMYR antagonist AC187, in contrast to agonists, increases alcohol intake in male rats (56). A limitation with the AMYR agonists such as sCT and AMY1213 compared to GLP-1R agonists, is that a tolerance toward treatment is observed (56, 57). As such, other agonists of AMYR should be tested in these drinking models in rats of both sexes. Just like the GLP-1 pathway, AMYR modulates other alcohol drinking behaviors. Indeed, acute administration of sCT prevents relapse drinking after a withdrawal period and reduces alcohol drinking in the operant self-administration model in alcohol-experienced male rats (55, 56).

#### 2.1.3. Ghrelin

As ghrelin is an orexigenic peptide, GHSR antagonists in contrast to GLP-1R or AMYR agonists are used to suppress alcohol intake (Figure 1). On this note, acute or repeated treatment with GHSR antagonists lowers alcohol intake in male animals consuming alcohol for long- (58, 59) or short-periods of time (60–64). Similarly, GHSR knockout rodents display a lower alcohol intake compared to their wild-type litter mates (58, 65). The decline in alcohol drinking may involve a GHSR in NAc, as female rats with a long-term alcohol exposure decreases alcohol drinking after local infusion into this area (66). It should be noted that the dose-dependent decline induced by the GHSR antagonist is more profound in rats exposed to alcohol for seven compared to 3 months (59). This indicates that GHSR antagonist treatment may be particularly suited for patients with a severe AUD which may respond better than a mild AUD or social drinkers; something that should be tested in future human studies. A clinical trial should also observe the possible lack of apparent tolerance toward GHSR antagonism treatment, a finding evident in male rats (59). On a similar note, no tolerance development is seen for agonists of the GLP-1R, but is evident AMYR.

Besides these profound effects on alcohol drinking, GHSR antagonists modulate other alcohol drinking behaviors. This can be observed in the operant self-administration paradigm, where the alcohol consumption and alcohol-seeking is diminished by pharmacological or genetical suppression of the GHSR (61, 65, 67). Moreover, GHSR antagonism suppresses relapse drinking after prolonged periods of alcohol abstinence (58, 59).

For GLP-1 and amylin studies, agonism and antagonism of the receptor have opposite effects; a finding also true for the ghrelin system. Indeed, in contrast to GHSR suppression ghrelin administration into the brain profoundly increases alcohol intake in male mice (58). However, the treatment outcome after peripheral systemic ghrelin administration varies. Thus, systemic administration of ghrelin has been shown to elevate alcohol intake in one study (68), but had no effect in another study (69). The role of ghrelin for alcohol intake is further debated as neutralization of circulating ghrelin does not affect alcohol consumption (70) and blood ghrelin levels are similar in high- and low-alcohol consuming rats (67).

### 2.2. The role of glucagon-like peptide-1, amylin, and ghrelin on alcohol-induced behaviors and neurochemistry

The findings that the rewarding aspects of alcohol enhances the risk of AUD diagnosis later in life (5) and that they are also important for several phases of the AUD cycle (4), indicate that reward is a central part of the addiction process. The reviewed studies collectively show that the three gut-brain peptides modulate alcohol-induced behaviors and neurochemistry, in which the mesolimbic system is one central neurocircuit (Figure 1). The ability of alcohol to activate the mesolimbic dopamine system and tentatively cause reward can be modeled in animals, where a locomotor stimulation and dopamine release in NAc are the central test [for review see (6, 7)]. Supportively, in humans the euphoria by alcohol correlates to the release of dopamine in NAc [for review see (7)]. Another animal model is the CPP test, which can be designed to reflect reward (rCPP) as well as memory of reward (mCPP) [for review see (6, 7, 71)]. On this note, the rewarding properties of alcohol as measured by the above-mentioned preclinical models are modulated by each of the three gut-brain peptides reviewed herein.

#### 2.2.1. Glucagon-like peptide-1

When it comes to the GLP-1 system, both Ex-4 and liraglutide have been found to block the ability of alcohol to activate the mesolimbic dopamine system. Indeed, systemic administration of either of the GLP-1R agonists block the hyperlocomotion and dopamine release in NAc caused by alcohol (41, 44). Moreover, two independent studies reveal that Ex-4 blocks the alcohol reward in the rCPP test (41, 42). Another aspect influenced by the GLP-1 pathway is the memory consolidation of alcohol reward, a behavior important when alcohol drinking transitions into AUD. When tested in the mCPP test both Ex-4 and GLP-1, but not liraglutide, suppresses this alcohol-related behavior (41, 42, 44). The rational for the discrepancy in these three GLP-1R agonists to influence mCPP are unknown, but may lay in different abilities to act in the brain or activate different downstream signaling pathways.

#### 2.2.2. Amylin

Similar to GLP-1 agonists, activation of AMYR blunts the alcohol-related behavioral and neurochemical responses. It was initially shown that sCT prevents the alcohol-induced hyperlocomotion, dopamine release in NAc and CPP-reward (55). The blunted hyperactivity after alcohol is also evident after sub-chronic pre-treatment with sCT, an effect correlating to decreased dopamine turnover and enhanced serotonin turnover in the VTA (72). On a similar note, acute administration of sCT at the end of the CPP test attenuates the memory of alcohol reward in the CPP test (55).

#### 2.2.3. Ghrelin

This suppression of alcohol-related behaviors and neurochemistry is also evident after manipulation of the ghrelin pathway. Specifically, the ability of alcohol to cause a locomotor stimulation, dopamine release in NAc and rCPP, is attenuated after either genetic or pharmacological suppression of the GHSR (58, 73). Furthermore, a GHSR antagonist attenuates the memory of alcohol reward in the CPP test (58). Besides acute treatment with a GHSR antagonist, sub-chronic pretreatment blocks the alcohol-induced locomotor stimulation in male mice, and effect not involving changed GHSR expression levels (73). As for alcohol drinking, the role of ghrelin itself for alcohol responses is further unclear as general knockout of ghrelin and neutralization of circulating ghrelin results in different alcohol outcomes. While male ghrelin knockout mice show an attenuated hyperlocomotion, NAc-dopamine and rCPP after alcohol (60, 74), this alcohol-induced activation of the mesolimbic dopamine system is unaltered by neutralization of circulating ghrelin (70).

### 2.3. Brain regions important for glucagon-like peptide-1, amylin, and ghrelin to modulate alcohol-related responses

As summarized above, either glucagon-like peptide-1, amylin or ghrelin modulate alcohol consumption patterns as well as alcohol-induced behaviors and neurochemistry, and the brain regions central for this interaction is to some extent mapped (Figure 2). Although the GLP-1R, AMYR, and GHSR expression is wide-spread, receptors within the brain rather than body appear central for their modulation of alcohol-mediated effects. Indeed, studies from promotor-specific GLP-1R knockout mice indicate that Ex-4 acts *via* the brain rather than body to modulate alcohol drinking (50). Similarly, circulating ghrelin does not appear to modulate the behavioral responses of alcohol (70). When it comes to important brain regions modulating this, there is some overlap as well as differences between the three peptides. NAc, VTA and laterodorsal tegmental area (LDTg) appear to be three important areas for this interaction. On this note, systemic administration of fluorescently marked Ex-4 or sCT is noted within these areas (75), indicating that these peptides enter through the blood-brain barrier and reach deeper brain regions. Importantly, GLP-1R, AMYR and GHSR are all expressed within these reward-related nuclei (16, 33–40, 76, 77).

#### 2.3.1. Nucleus accumbens

Nucleus accumbens is one relevant area as it is important for alcohol-induced reward and for the motivation to consume alcohol. When Ex-4 is infused into the NAc, the rodents don’t display an alcohol-induced hyperactivity or mCPP (78) and their alcohol intake is lowered (66, 78, 79). This interaction is further evident when comparing the NAc-GLP-1R expression of rats consuming different amounts of alcohol. Indeed, its expression is elevated in high- compared to low- alcohol preferring male rats (78). An association also found when it comes to the amylin system, as components of the AMYR are changed when comparing high- and low- alcohol preferring rats (56), Moreover, activation of AMYR in NAc by sCT attenuates both the behavioral and neurochemical responses to alcohol in male mice (80). A similar outcome is observed when it comes to the ghrelin pathway. Specifically, when infused into NAc the GHSR antagonist JMV2959 reduces alcohol intake in female rats (66). Moreover, the GHSR genes expression is higher in high- compared to low-alcohol preferring rats (40). A role of ghrelin in NAc for alcohol responses is also noted in human studies as the plasma levels of ghrelin are positively associated with ventral striatum (containing NAc) reactivity induced by cued alcohol craving in humans (81).

#### 2.3.2. The ventral tegmental area

The VTA is another area of interest as it is the main hub for the dopaminergic neurons of the mesolimbic dopamine system. As VTA is a heterogenous area, different sub-parts thereof may modulate alcohol-mediated behaviors differently. Indeed, Ex-4 infusion into the posterior part of the VTA decreases both the intake (42, 79) and operant self-administration (82) of alcohol. The role of GLP-1R in the anterior part of the VTA appear more complex as high doses of Ex4 into this sub-part reduces alcohol intake (83) whereas a lower Ex-4 dose does not alter the acute and chronic alcohol-mediated behaviors (78). While the role of the posterior part has not been elucidated when it comes to amylin or ghrelin, the anterior part appears central for both these peptides to influence alcohol drinking. Specifically, sCT into this sub-region attenuates alcohol-induced hyperlocomotion and dopamine release in NAc, as well as lowers alcohol intake (80). Moreover, ghrelin infusion into the anterior VTA elevates alcohol drinking (58, 83), and induces reward-related behaviors such as hyperlocomotion and dopamine release in NAc (73, 84–88). This interaction is further evident as low alcohol drinking rats have a lower expression of VTA-GHSR compared to high consuming rats (59), although this finding has not been replicated in humans (89). *Ex vivo* studies reveal that both dopamine and serotonin release in the VTA may deserve some extra interest in future studies as both by sCT and ghrelin alters these monoamines in this area (57, 90).

#### 2.3.3. Laterodorsal tegmental area

A third common area appears to be the LDTg, an area that projects to the VTA and modulates the activity thereof [for review see (91)]. Supportively, Ex-4 infusion into this area blocks the alcohol-induced locomotor stimulation, dopamine release in NAc, and prevents the memory of alcohol reward (mCPP) in male mice (78). Moreover, in male rodents with long-term alcohol exposure Ex-4 infusion into the LDTg reduces alcohol intake (78). These findings are to some extent similar after AMYR activation. Indeed, the ability of alcohol to cause a hyperlocomotion, dopamine elevation in NAc and alcohol drinking is reduced after sCT into LDTg (80). On the contrary, this does not converege into the memory of alcohol resard as sCT into LDTg does not block the mCPP (80). LDTg also appear important area for ghrelin to act, as the intake of alcohol and the activity of the VTA-dopamine neurons is enhanced after ghrelin infusion into the LDTg of male rodents (58, 84).

#### 2.3.4. Other tentative brain areas

Besides NAc, VTA, and LDTg, additional areas may be of interest for the alcohol and gut-brain peptide interaction. Some of these have been pinpointed in relation to GLP-1 and ghrelin, but not for amylin. Lateral hypothalamus appears central for both GLP-1 (79) and ghrelin (92) to influence alcohol-mediated behaviors. Moreover, GLP-1R agonists acting in the hippocampus, lateral habenula and NTS modulate alcohol responses, whereas ghrelin in the amygdala appears to have such effect (51, 92–97).

### 2.4. Human studies displaying an interaction between alcohol and the pathways of either GLP-1 or ghrelin

Some human studies have explored the role of GLP-1 or ghrelin pathways for alcohol intake in humans, whereas no such studies on amylin are available yet.

#### 2.4.1. Glucagon-like peptide-1

In one early pilot study, diabetic patients treated with liraglutide display lower alcohol intake compared to those treated with non-GLP-1 compounds (98). These findings are supported by data from a recent RCT exploring the effect of the GLP-1R agonist exenatide on alcohol intake in patients with AUD (99). While exenatide does not alter consumption in AUD patients with a normal weight, it substantially lowers alcohol intake in overweight patients with AUD (99). In further support for the interaction between alcohol and GLP-1R are the human genetic data revealing an association between GLP-1R polymorphisms and AUD diagnosis and high alcohol intake (48). It should, however, be noted that the association between polymorphisms of the GLP-1R gene and AUD was not replicated in a different cohort (100). In contrast to ghrelin, studies exploring the relationship between the plasma levels of GLP-1 and alcohol is studied to a lesser extent. Indeed, one recent human study reveals that the plasma levels of GLP-1 is lower after alcohol consumption (93).

#### 2.4.2. Ghrelin

When it comes to ghrelin, clinical studies show that the craving for alcohol (101) and hangover due to intravenous alcohol administration (102) are reduced by the inverse GHSR agonist PF-5190457. Conversely, intravenous ghrelin enhances alcohol craving and decreases the latency to the first intravenous alcohol infusion in patients with AUD (95, 103). The human genetic data revealing an association between polymorphisms of the pre-pro-ghrelin or GHSR (104–108) genes and different aspects of alcohol drinking and AUD diagnosis provide further support for this ghrelin-alcohol interaction.

Circulating ghrelin is associated with some but not other aspects of AUD [for review (12)]. However, the findings that plasma levels of ghrelin are positively associated with alcohol craving (81, 109–112), possibly *via* the ventral striatum (81), may be of extra interest as they correspond with the findings that ghrelin increases alcohol craving in AUD patients (95, 103). On a similar note, elevated circulating ghrelin is positively associated with the subjective intensity of alcohol (113). The interaction between plasma ghrelin and AUD parameters are extensively reviewed elsewhere [for review (12)].

#### 2.4.3. Future directions and summary

Collectively, these preclinical and clinical studies show that the GLP-1, amylin and ghrelin pathways modulate alcohol intake and alcohol responses (behavioral and neurochemical). They further suggest that the ability of these gut-brain peptides to reduce alcohol drinking possibly involves an attenuated alcohol reward.

However, when interoperating the above summarized data a wide range of confounding factors should be taken into consideration. One of these are a general effect on ingestion or caloric intake since the gut-brain peptides control feeding. However, this appears less likely as Ex-4 also decreases alcohol intake when the oral route is avoided and alcohol is administered intravenously instead (114). Besides, each of the tested gut-brain peptides modulates the behavioral and neurochemical responses induced by additive drugs as well as drug-taking for other drugs than alcohol (for example nicotine, cocaine, and amphetamine), which are not influenced by ingestion or calories [for review see (115)]. Moreover, sCT reduces alcohol drinking without affecting the intake of peanut butter or a highly palatable chocolate-flavored beverage (55, 56). Receptor non-selectivity is another aspect that may influence the obtained data as sCT in addition to AMYR activates CTR. However, this seems unlikely as the different pharmacological agents targeting one receptor (GLP-1R, AMYR or GHSR) display a similar ability to reduce alcohol drinking. Moreover, the outcome of an agonist of one receptor (GLP-1R, AMYR or GHSR) show the opposite results as the antagonist of the same receptor (45, 56, 58). On a similar note, the GLP-1R antagonist Ex9 blocks the ability of Ex-4 to reduce alcohol drinking (45). Enhanced metabolism of alcohol is another possibility that could influence the obtained data. However, studies have revealed that neither GLP-1R or AMYR agonists changes the blood alcohol levels after systemic administration of alcohol (44, 55). As AMYR agonists does not alter corticosterone in the plasma, stress appears as a less likely confounding factor for that peptide (55, 56). In terms of GLP-1R signaling, the doses that blocks the alcohol-mediated behaviors do not alter corticosterone in plasma (116, 117). However, as other doses of GLP-1, Ex-4 and liraglutide do affect plasma corticosterone (118) the influence of stress should be considered as a tentative confounding factor. Similarly, ghrelin is connected to stress response and the hypothalamus-pituitary-adrenal axis (119, 120). As such, the interaction with stress is an important consideration for future studies regarding ghrelin’s effects on alcohol. Nausea is another factor that tentatively influences the obtained results since it is induced by GLP-1R agonists (121). Moreover, GHSR antagonist could tentatively be aversive as ghrelin reduces nausea (122). However, this appears unlikely as the dose-range of the tested pharmaceutics does not cause aversion, malaise or nausea (27, 33, 37, 121, 123, 124). On a similar note, neither of the tested pharmaceuticals causes a preference *per se* in a CPP test, indicating that they do not condition for aversion (123). In further accordance, the tested agents (i) increase, rather than decrease, and water intake, (ii) block alcohol-induced hyperlocomotion and dopamine release in NAc which are parameters unaffected by aversion, and (iii) attenuates drug-related responses that are driven by reward rather than by malaise [for review see (115)]. To date, the three peptides have been shown to modulate various alcohol consumption patterns, alcohol-induced behaviors and neurochemistry, and thus have been suggested to regulate aspects of the AUD cycle. It should, however, be considered a limitation that one animal model cannot reflect all aspects of AUD seen in humans.

Despite the fact that extensive preclinical and to some extent clinical literature support the contention that gut-brain peptide modulate alcohol responses additional experiments should explore this further. Based on these initial preclinical studies the hypothesis that GLP-1, amylin, and ghrelin controls alcohol drinking behaviors though their ability to suppress alcohol-induced reward has been formed. However, if this is true and whether this translates to a human situation needs to be addressed in future studies. Another area of interest is how these gut-brain peptides modulate different aspects of the AUD cycle. In contrast to the GLP-1 pathway, that suppresses abstinence symptoms during withdrawal, the role of amylin or ghrelin systems for such behaviors remains to be explored. Although females have been included in a few studies (47, 57, 66), additional preclinical studies should compare the outcome between sexes and define underlying differences and similarities. This contributes to further understanding of the AUD pathophysiology that diverges between gender [for review see (125)]. Moreover, mechanisms of action should be defined for each peptide in detail, where monoaminergic signaling in the VTA or GABAergic neurotransmission appear interesting for the tested gut-brain peptides (32, 47, 57, 72, 90, 126–132). It should also be emphasized that the brain regions and neurocircuits responsible for the ability of gut-brain peptide to modulate alcohol responses most likely overlap to some extent, but most likely diverge. Moreover, the ability of different agonists of each receptor to influence alcohol responses may differentiate, another focus on warranted studies. Another aspect that deserves more attention is the role of each step of the pathways (i.e., precursor, enzymes, and metabolites) for the alcohol responses.

Although these peptides act separately to control alcohol drinking, studies have revealed that they act synergistically to reduce feeding and body weight (133). As AUD has a heterogenous pathophysiology, the possibility that combination treatments act synergistically to reduce alcohol intake should be considered and explored in up-coming studies. Moreover, the possibility that the treatment design, i.e., how and when these compounds are combined, influences alcohol drinking should be explored in up-coming studies. Another way to influence alcohol drinking is dietary alterations including kerogens which are known to influence the circulating levels of these gut-brain-peptides (134). In line with this suggestion, ketogenic diets lower alcohol intake (135) and abstinence symptoms (135, 136).

As some initial studies show in interaction between alcohol responses and GLP-1 and ghrelin, but not amylin, additional human studies exploring these associations warranted for the future. These included, but are not limited to (i) plasma associations studies, (ii) human genetic studies, and (iii) laboratory studies investing the interaction toward consumption, subjective experiences and craving. On a final note, clinical studies should further explore the influence of these gut-brain peptides on alcohol intake and alcohol craving in patients with AUD with a comorbid obesity or smoking diagnosis.

## Author contributions

EJ wrote the first draft of the manuscript. MT-A and OS wrote the final version of the manuscript and thus contributed significantly to the summary of data, confounding factors, and future directives. All authors approved the final version of the manuscript.
